# The roles of exosomes in the pathogenesis and treatment of coronary heart disease with depression and/or anxiety

**DOI:** 10.1016/j.bbih.2025.101113

**Published:** 2025-09-19

**Authors:** Jiecheng Huang, Ying Piao, Xin Jiang, Jingjin Liu

**Affiliations:** a The Second Clinical Medical College, Jinan University, Shenzhen, 518020, Guangdong, China; b Department of Radiation Oncology, Shenzhen People's Hospital (The Second Clinical Medical College, Jinan University; The First Affiliated Hospital, Southern University of Science and Technology), Shenzhen, 518020, Guangdong, China; c Department of Geriatrics, Shenzhen People's Hospital (The Second Clinical Medical College, Jinan University; The First Affiliated Hospital, Southern University of Science and Technology), Shenzhen, 518020, Guangdong, China; d Shenzhen Clinical Research Center for Geriatrics, Shenzhen People's Hospital, Shenzhen, China

**Keywords:** Exosomes, Coronary heart disease, Depression, Anxiety, Pathogenesis, Treatment

## Abstract

Coronary heart disease (CHD) patients have been found to also possess high anxiety and depression rates, which have been considered as significant risk factors for the disease. One possible underlying biological mechanism behind anxiety/depression being associated with CHD may be exosomes, extracellular vesicles produced by cells throughout the body. These exosomes contain various proteins and miRNAs that could exert a variety of physiological and pathological effects. However, the precise role they play in CHD with anxiety/depression has still not been fully elucidated. In this review, we summarized the current research on exosome involvement in the pathogenesis of CHD with anxiety/depression, particularly focusing on inflammatory responses, neuroendocrine signaling, sympathetic nervous system (SNS) regulation, platelet activation, and endothelial injury. In particular, for inflammatory responses, exosomes have been associated with increased pro-inflammatory cytokine release, such as interleukin (IL)-1β, while for neuroendocrine signaling, the miRNAs miR-135a-5p and miR-320a have been implicated in increasing glucocorticoid signaling. As for SNS regulation, exosome miRNAs are involved in downregulating Nrf2, leading to increased sympathetic nerve excitation, while inhibiting exosome production counteracts platelet activation, in turn lowering thrombosis risk for CHD. Endothelial dysfunction could be promoted by exosomes carrying miR-155. On the other hand, exosome contents exert beneficial effects that could be used for treatment strategies, such as miR-1246 alleviating hypoxia-induced myocardial tissue damage, as well as miR-188–3p lowering nigrostriatal autophagy. Overall, identifying the roles that exosomes play in CHD with concurrent anxiety/depression pathogenesis, as well as potential alleviation, may be greatly beneficial for formulating effective treatment strategies.

## Highlights

• Anxiety and depression are important independent CHD risk factors.
• Exosomes can contribute to the pathogenesis and treatment of coronary heart disease with depression and/or anxiety.
• Exosomes maybe play key roles in pathogenesis and developing potential treatment approaches.

## 1 Introduction

Coronary heart disease (CHD) is a common type of heart disease, caused by impaired blood flow through the coronary arteries. It is associated with significant medical and economic burdens worldwide, owing to its widespread prevalence, along with high morbidity and mortality rates. In recent years, CHD patients have been found to have higher anxiety and depression rates (Sohier et al., 2024), such as in an epidemiological study showing that depression prevalence was 15–30 % higher in CHD patients, than in those without (Penninx, 2017). Based on these observations, anxiety and depression, which are common mental disorders, have been considered to be significant risk factors for CHD; indeed, they have been found to be strongly linked to its incidence and prognosis (Vicario and Cerezo, 2023). For instance, in some studies, CHD patients with anxiety/depression demonstrated significantly higher morbidity and mortality risks than those who did not (Win et al., 2011; Watkins et al., 2013), and a meta-analysis noted that individuals with clinical depression was 2.5 times more at risk for myocardial infarction (MI) or coronary death, compared to the general population (Rugulies, 2002). In light of these findings, researchers have started to further elucidate the role of depression and anxiety disorders in CHD (Li et al., 2024). As a result, treating psychological conditions, such as anxiety and depression, could aid in improving patient symptoms and quality of life, particularly with respect to CHD.

However, the exact mechanisms linking anxiety/depression to increased CHD risk are complex, multifactorial, and still not fully clear, though they cannot be purely attributed to adverse lifestyle behaviors and traditional CHD risk factors. Therefore, fully elucidating CHD characteristics and pathogenesis, in anxious/depressed patients, could aid in developing treatment strategies for them. Nevertheless, current studies have shown that several mechanisms, such as neurobiological changes, emotional stress, autonomic dysfunction, and genetic predispositions, may be involved in the relationship between anxiety/depression and CHD (Vaccarino et al., 2020). One potential underlying molecular mechanism contributing to the pathological development of CHD with anxiety/depression is exosomes. Exosomes are an extracellular vesicle subtype, being ∼40–150 nm in diameter (Jeppesen et al., 2019), with a lipid bilayer structure (Zaborowski et al., 2015). They are secreted by various cell types, leading to them being widely distributed in numerous bodily fluids, such as in plasma and urine (Doyle and Wang, 2019). Their contents include proteins, DNA, non-coding RNAs (ncRNAs), etc., making exosomes natural carriers for a variety of signaling molecules, and enabling them to play key roles in intercellular communication and signal transduction (Gong et al., 2024). Owing to those properties, exosomes have become a topic of great interest for cardiovascular disease research, such as CHD with anxiety/depression. This review summarizes the current research on the roles that exosomes play in CHD with anxiety/depression, as well as potential exosome-based treatment strategies for the disease.

## 2 The roles of exosomes in the pathogenesis of CHD with depression/anxiety

**Fig. 1:**
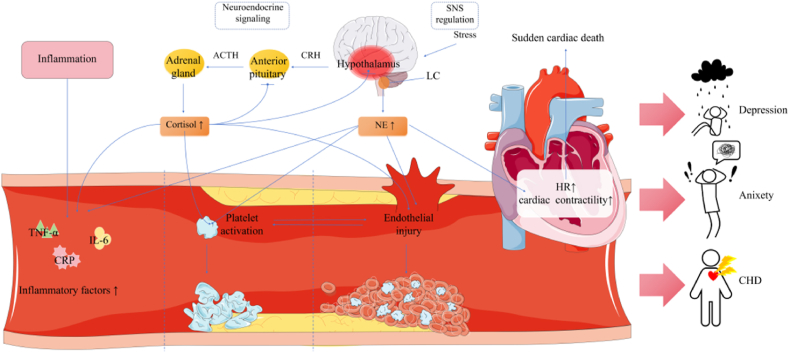
The pathogenesis of CHD with depression/anxiety include inflammatory responses, neuroendocrine signaling, sympathetic nervous system regulation, platelet activation, and endothelial injury.Inflammation activates immune cells to produce a variety of inflammatory factors, such as TNF-α, IL-6 and CRP.HPA activation begins with stress triggering the hypothalamus to release CRH, which activates the anterior pituitary to secrete ACTH; ACTH acts on the adrenal cortex to promote cortisol synthesis, which inhibits CRH and ACTH in a negative feedback loop.Stress causes the amygdala to activate the central SNS, in turn activating the LC in the brainstem, which increases levels of NE. The plasma NE levels were associated with high cardiac sympathetic activity. Moreover, the high levels of cortisol and NE affect the function of vascular endothelial cells and platelet. The mechanisms play key roles in CHD, anxiety and depression. TNF-α = Tumor necrosis factor-α; IL-6 = Interleukin-6; CRP=C-reactive protein; CRH = corticotrophin-releasing hormone; ACTH = adrenocorticotropin hormone; SNS = sympathetic nervous system; LC = locus coeruleus; NE = norepinephrine; HR=Heart Rate; CHD=Coronary heart disease.

The pathogenesis of CHD with depression/anxiety has not been fully described, though multiple processes have been proposed to be involved, including inflammatory responses, neuroendocrine signaling, sympathetic nervous system (SNS) regulation, platelet activation, and endothelial injury. These processes have been considered to be important pathological mechanisms linking CHD and anxiety/depression, the latter of which have been noted to be closely related to the exacerbation of CHD symptoms and poor prognoses. With respect to inflammatory responses and neuroendocrine signaling, inflammation and excess hypothalamus-pituitary-adrenal (HPA) axis activity have been closely associated with cardiac dysfunction, depression and anxiety-like behaviors. As for SNS regulation, abnormal SNS activities lead to the over-stimulation of the secretion for some neurotransmitters, such as norepinephrine (NE), which affects vascular endothelial (EC) and smooth muscle cell function, and subsequently contributes to atherosclerosis pathogenesis. Additionally, depression and anxiety symptoms have been linked to platelet activation and rapid accumulation, as well as endothelial dysfunction, in turn accelerating disease progression. All of these pathophysiological mechanisms are summarized in Fig. 1, and exosomes have been observed to play roles in all of them (Table 1). This is owed to exosomes serving as the most prominent intercellular communication mediator. More specifically, exosomal miRNAs have been observed by Jiao et al. to promote inflammatory damage in sepsis (Jiao et al., 2020); by contrast, in astrocytes, exosome downregulation by pro-inflammatory cytokines reduces their neuroprotective effects (Jin et al., 2024). Exosome counts have also been linked to cortisol concentrations, which itself stem from HPA axis activity, by Colitti et al., (2019). Furthermore, Tian et al. noted that circulating exosomes promoted SNS activation (Tian et al., 2022), while Li et al. found that they induced endothelial injury (Li et al., 2023). Overall, exosomes play numerous roles in these physiological processes, and alterations in their secretion and contents contribute to the progression of CHD with depression/anxiety. The following sections elaborate each proposed mechanism in detail.

**Table 1:** Studies of exosomes in the pathogenesis of coronary heart disease with depression/anxiety.

Author	Study Model	Main Findings
Tu et al. (2022)	Animal experiment	Exosomes are associated with changes in inflammatory cytokines, thereby playing roles in inflammatory progression.
Jiao et al. (2020)	Animal experiment	miRNAs from exosomes promote inflammatory damage in sepsis and subsequent organ injury.
Jin et al. (2024)	Animal experiment	Pro-inflammatory cytokines lower neuroprotective effects by down-regulating astrocyte exosomes
Colitti et al. (2019)	Animal experiment	Increasing cortisol levels are involved in changes in various miRNAs.
Zekas et al. (2019)	Observational study	Total exosome counts are associated with cortisol concentrations.
Tian et al. (2022)	Animal experiment	Circulating exosomes promote oxidative stress and sympathetic nerve excitation.
Li et al. (2023)	Animal experiment	Exosomes induce endothelial injury and barrier dysfunction.

### 2.1 Inflammatory responses

Inflammation is an important pathological mechanism for CHD, in which studies have shown that it plays an important role in atherosclerotic lesion occurrence and progression. More specifically, low-density lipoprotein deposition and oxidation in the arterial intima, along with endothelial injury and hemodynamics changes, trigger inflammatory responses and activate endothelial cells via numerous complex mechanisms (Hansson, 2005). These activated endothelial cells subsequently produce adhesion factors and chemokines, which promote immune cell aggregation and activation (Saxena et al., 2016). Activated immune cells, in turn, produce a variety of inflammatory cytokines, eventually leading to plaque rupture and thrombosis (Montecucco et al., 2017). In particular, studies by Kucharz and Wilk, as well as Karpinski et al., found that acute MI patients had significantly higher interleukin-6 (IL-6), IL-10, and C-reactive protein (CRP) levels than control (Kucharz and Wilk, 2000; Karpinski et al., 2009). With respect to CHD, Su et al. observed that serum IL-6 was significantly associated with all-cause mortality and cardiovascular mortality in hospitalized CHD patients (Su et al., 2013), while Arbel et al. identified that the neutrophil/lymphocyte ratio was independently associated with CHD severity and worse 3-year outcomes (Arbel et al., 2012). All these findings therefore indicate that CHD was associated with higher inflammation.

Inflammation has also been linked with anxiety and depression. For instance, depression has been associated with systemic immune activation, including inflammatory markers, immune cell numbers, and antibody titer abnormalities (Beurel et al., 2020). In fact, compared to control, patients with depression have elevated levels of inflammatory factors (Mikova et al., 2001; Miller et al., 2002; Lindqvist et al., 2017), and elevations of those cytokines were positively correlated with depression severity (Renna et al., 2018). Similar findings were found for anxiety, in which patients with anxiety disorders also had increased pro-inflammatory cytokine levels, compared to control, and those increased levels positively correlated with anxiety severity (Vogelzangs et al., 2013; Hou et al., 2017; Zou et al., 2020). Therefore, inflammatory reactions could trigger, as well as worsen, anxiety/depression.

Considering that CHD and anxiety/depression both were aggravated by inflammation, inflammatory responses could serve as an important mechanism behind the interactions between the 2 diseases. Indeed, the anxiety/depression-associated inflammatory response has been found to be closely related to CHD and other adverse cardiovascular events, as observed by Steptoe et al., in which white blood cell counts in acute coronary syndrome patients is associated with increased anxiety scores, as well as being possibly related to cognitive symptoms of depression (Steptoe et al., 2013). Additionally, Sforzini et al. found that high-sensitivity CRP (hsCRP), an inflammatory biomarker, was positively correlated with depressive symptom severity in CHD patients (Sforzini et al., 2019), and Pizzi et al. identified that depression positively correlated with intima-media thickening (IMT; (Pizzi et al., 2010). These associations between inflammatory responses with CHD and anxiety/depression are likely owed to inflammatory signaling pathways, such as nuclear factor kappa B (NF-κB). NF-κB has been found to be involved in immune and inflammatory activities, along with playing an essential role in EC activation and pathological angiogenesis, all of which are involved in CHD progression (Maracle et al., 2018). In fact, numerous pro-inflammatory stimuli, such as TNF-α, IL-1β, and oxidized lipoproteins, activate the endothelial inflammatory cascade via NF-κB (Maracle et al., 2018), while Su et al. observed that inhibiting TLR4/MyD88/NF-κB signaling with nicorandil ameliorated lipopolysaccharide (LPS)-induced myocardial inflammation and improved cardiomyocyte survival (Su et al., 2018). Furthermore, sustained NF-κB hyper-activation dysregulates depression and anxiety-related immune signaling and inflammatory reactions in the central nervous system (Wang et al., 2023). By contrast, Wang et al. showed that transcutaneous auricular vagus nerve stimulation exerted anti-depression effects via α7nAchR/NF-κB signaling (Wang et al., 2021). Another prominent inflammatory signaling pathway is the NLR family pyrin domain containing 3 (NLRP3) inflammasome, a cytosolic multiprotein complex comprising innate immune receptor protein NLRP3, adapter protein apoptosis-associated speck-like protein containing a CARD, and inflammatory protease caspase-1. It has been closely associated with inflammatory responses (Huang et al., 2021), and aberrant inflammasome activation has been linked to disease pathogenesis, such as CHD (Jahromi and Rezaei, 2025). In fact, a meta-analysis noted that anti-inflammatory therapies, inhibiting the NLRP3/IL-1β/IL-6/CRP pathway, were able to prevent MI and coronary re-vascularization in CHD (Pan et al., 2025). The inflammasome has also been regarded as an essential process behind depression development (Alcocer-Gómez et al., 2016), and its activity was able to be inhibited by antidepressants (Alcocer-Gómez et al., 2017), suggesting that inflammasome inhibitors could serve as an anti-depression/anxiety strategy (Xia et al., 2023). All of these observations therefore indicate that inflammatory reactions are important pathological mechanisms contributing to CHD and anxiety/depression.

Exosomes have also been identified in a large number of studies to be involved in the inflammatory response process, suggesting that they may play roles in disease progression. Owing to their close association with inflammatory and other pathological processes, their content and composition could change throughout disease development, such as for sepsis (Yuan et al., 2024). More specifically, a study showed that exosomes from sepsis patients were associated with increased miR-885–5p, IL-1β and IL-18, as well as cardiomyocyte pyroptosis, along with decreased homeobox-containing protein 1 (HMBOX1) (Tu et al., 2022), which is an important immunosuppressive factor that curbs inflammatory responses (Zhao et al., 2018). HMBOX1 has also been found to regulate vascular endothelial cell autophagy and apoptosis (Ma et al., 2019). Additionally, Jiao et al. observed that miR-15b-5p and miR-378a-3p from platelet-derived exosomes were involved in inducing neutrophils to form neutrophil extracellular traps (NETs), via the Akt/mTOR autophagy pathway, thus promoting inflammatory damage (Jiao et al., 2020). Furthermore, Sui et al. showed that LPS could increase exosome and pro-inflammatory cytokine release from mouse alveolar macrophages (Sui et al., 2021), while Xu et al. noted that miR-155–5p from macrophage-derived exosomes could drive extensive macrophage activation and M1 polarization through the MSK1/p38-MAPK axis, thereby inducing inflammation and tissue damage (Xu et al., 2023). On the other hand, Jin et al. found that pro-inflammatory cytokines can reduce the neuroprotective effect of exosomes by down-regulating astrocyte exosomes (Jin et al., 2024). Exosomes could also aid in suppressing the pro-inflammatory activities of the NF-κB signaling pathway, such as Lee et al. showing that they could serve as delivery vehicles for NF-κB inhibitors to treat neuroinflammation (Lee et al., 2025), as well as Ouyang et al., who observed that bone marrow mesenchymal stem cell (BMSC)-derived exosomes alleviated MI by inhibiting NF-κB to promote M2 macrophage polarization (Ouyang et al., 2024). Likewise, adipose-tissue derived MSC-produced exosomes was able to attenuate atherosclerosis in low-density lipoprotein receptor-deficient mice, via inhibiting MAPK and NF-κB signaling in ECs and macrophages, along with activating macrophage STAT3 signaling (Takafuji et al., 2019). Consequently, alterations in exosome secretion and contents are closely associated with inflammatory progression and tissue damage, leading to exosomes being considered as a potential therapeutic agent for CHD and depression/anxiety. Owing to inflammation being a shared underlying mechanism behind CHD and depression/anxiety, anti-inflammatory applications of exosomes could alleviate these diseases.

### 2.2 Neuroendocrine signaling

A key component of neuroendocrine signaling is the HPA axis, which regulates a variety of bodily activities, as well as coordinates interactions between hormones, glands and some midbrain regions. Activation begins with stress triggering the hypothalamus to release corticotrophin-releasing hormone (CRH), which activates the pituitary gland to secrete adrenocorticotropin hormone (ACTH). ACTH acts on the adrenal cortex to promote glucocorticoid synthesis, which inhibits CRH and ACTH in a negative feedback loop. HPA axis dysregulation could result in physiological dysfunction, such as elevated cortisol levels, in turn contributing to cardiac dysfunction (Yang et al., 2024; Morbach et al., 2024; Faresjo et al., 2024). The HPA axis has also been found to be closely related to anxiety and depression occurrence and development, as it plays important regulatory roles in maintaining homeostasis and stress responses. More specifically, chronic stress responses and depression could cause continuous HPA axis activation. This results in impaired immune system function and the development of certain pathologies, including increased NK and cytotoxic T cell activities, as well as phagocytosis and pro-inflammatory cytokine production, all of which could compromise immune responses (Reiche et al., 2004). Indeed, excess HPA axis activity has been associated with anxiety and depression, as sustained activation leads to increased plasma cortisol and ineffective negative feedback inhibition (Juruena et al., 2020). This may be due to alterations in the subgenual prefrontal cortex, an important structure for managing anxiety and emotions. It is significantly smaller in depression patients, resulting in persistent anxiety, continued HPA activation, and depressive behaviors, such as low mood, cognitive dysfunction, and anhedonia (Gold, 2015). Furthermore, Yu et al. found that depression in mice could activate the HPA axis, resulting in increased glucocorticoid levels, and in turn, changes in immune cell numbers and cytokine levels (Yu et al., 2025). Likewise, Zheng et al. found that mice, exposed to chronic social defeat stress to induce depression and anxiety, had increased serum CRH, ACTH, and corticosterone (Zheng et al., 2025). By contrast, ginseng ethanol extract, via regulating FKBP51 on the glucocorticoid receptor, strengthened HPA axis negative feedback and alleviated depressive symptoms (Li et al., 2024). Therefore, regulating HPA axis function and reducing corticosterone levels could reduce depression and anxiety.

With regards to neuroendocrine and stress signaling, exosomes play an important role in intercellular communication, and mediate brain-periphery communication (Makrygianni and Chrousos, 2023). More specifically, exosomes could mediate neuronal impulse transmission like traditional neurotransmitters, as well as intercellular transmission via their protein or miRNA cargo (Yu et al., 2025). Moreover, a study showed that in a mouse model, control and stressed mice had different neuronal exosome miRNA profiles, suggesting that they could be used as potential biomarkers for acute stress (Sung et al., 2021). Exosome miRNA expression could also be modulated to yield antidepressant effects, such as by n-3 polyunsaturated fatty acids (PUFA) to down-regulate miRNA-218, and subsequently, the HPA axis and pro-inflammatory cytokines (Choi et al., 2020). By contrast, n-3 PUFA up-regulated miRNA-182, which correlated with decreased hippocampus brain-derived neurotrophic factor (BDNF) expression, subsequently exacerbating depression-like behaviors (Li et al., 2016).

Exosomes have also been found in a number of studies to be involved in regulating the HPA axis and cortisol levels, such as by Colitti et al., who found that cows with elevated cortisol had differences in various exosome miRNAs, such as miR-2904-1, miR-142, miR-2284x and miR-30b-3p, compared to control. Functional enrichment analysis showed that exosome miR-135a-5p and miR-320a were involved in the glucocorticoid receptor signaling pathway (Colitti et al., 2019). Additional studies have also found that exosome numbers are significantly related to the levels of stress-related parameters, such as cortisol (Zekas et al., 2019; Perge et al., 2018). Therefore, exosomes, mainly via their miRNA contents, play an essential role in regulating HPA and cortisol levels; maintaining their homeostasis serves to act against depression/anxiety.

### 2.3 SNS regulation

SNS is an important component of the autonomic nervous system, in which sympathetic preganglionic fibers originate from neurons in the medio-lateral column of the spinal cord, and project to the paravertebral and prevertebral ganglia, where they connect with postganglionic neurons that in turn, project to target organs (Scott-Solomon et al., 2021). Sympathetic preganglionic neurons also synapse with adrenal chromaffin cells to stimulate epinephrine and NE production from the adrenal medulla, which enter the blood circulation and affect distant tissues (Wang et al., 2023). Furthermore, arteries may be innervated by sympathetic nerves, whose activity could, through multiple mechanisms, affect the function of vascular ECs and smooth muscle cells, as well as monocyte infiltration, all of which are involved in atherosclerosis pathogenesis, thereby leading to cardiovascular and cerebrovascular diseases. As early as 1984, Cohn et al. found that plasma NE levels were significantly associated with poor prognoses and risk of death in heart failure (Cohn et al., 1984). Additionally, high cardiac sympathetic activity is a risk factor for sudden cardiac death (Brunner-La Rocca et al., 2001), as well as poor heart disease prognoses, as confirmed by a large number of studies (Petersson et al., 2005; Schiller et al., 2015). Moreover, Parker et al. found that sympathetic nerve activity could affect drug metabolism in coronary artery disease (CAD) patients, thus affecting their therapeutic effects (Parker et al., 2020). Overall, unusual SNS activity is significantly related to cardiac dysfunction and poor prognoses.

SNS has also been found to be closely related to anxiety and depression, in which NE is an important neurotransmitter tightly associated with anxiety and depression symptoms. Stress causes the amygdala to activate the central SNS, in turn activating the locus coeruleus (LC) in the brainstem, the main NE synthesis site. Therefore, under stress conditions, CRH and the LC-NE system play major roles in regulating stress responses. In fact, CRH is not only associated with anxiety and stress-related behaviors, but could also increase tyrosine hydroxylase in LC. Tyrosine hydroxylase is the rate-limiting enzyme for catecholamine synthesis, thereby regulating NE release (Perrelli et al., 2024). Consequently, disturbing the noradrenergic system could lead to anxiety-depressive-like manifestations (McCall et al., 2015), as demonstrated in a zebrafish model, where exposure to polystyrene microplastics lowered their NE levels, in turn inducing depression-like signs (Yang et al., 2025). This was in line with the observations of Kurban et al., in which NE downregulation triggered depressive behaviors in rats (Kurban et al., 2024). With respect to anxiety, Gong et al. found that in chronic insomnia disorder patients, LC-NE functional changes, specifically abnormalities in the left dorsal anterior cingulate cortex, were associated with anxiety symptoms (Gong et al., 2021). However, NE was still found by Tillage et al. to be essential for expressing acute stress-induced anxiety, as NE-knockout mice, compared to wild-type, were unable to express normal anxiety-like behaviors (Tillage et al., 2021). Regardless, all these investigations thus demonstrated that SNS, via multiple regulatory mechanisms, plays key roles in anxiety and depression symptoms.

With respect to exosomes and SNS, exosome proteins, lipids, and nucleic acids could participate in regulating intercellular communication and paracrine signals. For instance, miRNA could be selectively upregulated in response to stress, and released from cells by exosomes, which serve as carriers for these biological signals. Tian et al. found that in post-MI rats, as well as humans with chronic heart failure, cardiac-derived miRNA that targeted Nrf2 was up-regulated and circulated by exosomes to the anterior rostral ventrolateral medulla of the sympathetic nerve, thereby mediating Nrf2 downregulation to promote oxidative stress and sympathetic nerve excitation (Tian et al., 2022). Exosomes have also been found to participate in heart-brain sympathetic neuroendocrine interactions, in which Beninson et al. found that acute stressors modulated protein and miRNA profiles within circulating plasma exosomes in rats. This demonstrates that stress responses can induce exosome cargo adjustments, which is mediated by SNS activation of α-1 adrenergic receptors (Beninson et al., 2014). Thus, exosomes play an essential role in stress reactions, via their regulation of intercellular communication.

### 2.4 Platelet activation

Platelets are activated during vascular injury by adhering to adhesion proteins (ex. von Willebrand factor, collagen) or soluble platelet agonists (ex. ADP, thrombin, thromboxane A2) (Li et al., 2010), resulting in a series of events, including shape changes, plus adhesion, aggregation, and release reactions, that all culminate in stable thrombus formation. Rapid adhesion and accumulation, post-platelet activation, is an important factor for CHD development, in which high on-treatment platelet activity (HPR) after percutaneous coronary intervention is associated with increased cardiovascular event incidence and mortality (Gawaz, 2016). A number of clinical studies have also shown that increased circulating platelet activation is associated with CAD severity and atherosclerosis progression (Gawaz et al., 2005; Langnau et al., 2021; Grande et al., 1990). Moreover, a close relationship exists between anxiety/depression and platelet activation, in which a study showed that anxiety symptom severity significantly correlates to serotonin-mediated platelet aggregation and activation in CHD (Wittstein, 2010). This is further supported by Aschbacher et al., who found that increased depression and anxiety symptoms were associated with prolonged SNS and significant platelet activation (Aschbacher et al., 2008), as well as Reid et al., who observed that mental stresses could induce platelet activation in CHD patients. Overall, psychological factors may be associated with adverse cardiac outcomes in those patients (Reid et al., 2009).

Platelets could also release exosomes, and the miRNAs within them could be involved in regulating vascular homeostasis, inflammation and platelet function, all of which are important processes in cardiovascular diseases. For instance, McManus and Freedman showed that high miR-126, miR-150, miR-197 and miR-223 expression in platelets and their micro-particles is closely related to MI risk (McManus and Freedman, 2015). Furthermore, a randomized controlled study found that nitrate supplementation reduced platelet-derived exosomes and platelet aggregation, along with inhibiting platelet activation, all of which lowered thrombosis risk in CAD patients (Burnley-Hall et al., 2018). Consequently, increased platelet activation could aggravate cardiovascular diseases, such as CHD, mainly through mediators released from exosomes.

### 2.5 Endothelial injury

ECs, which are biologically significant for regulating inflammatory responses, thrombosis and vascular tone, contribute to atherosclerosis and CHD pathogenesis via endothelial injury and inflammation. More specifically, vascular endothelial dysfunction could lead to imbalances in vasoactive substance release, namely lowered nitric oxide (NO), along with increased endothelin-1 (ET-1) and angiotensin II (Ang II), leading to aggravated vasoconstriction, thrombosis, and CAD progression. Aside from their cardiovascular effects, ET-1 also regulates cerebral vascular tone, thereby contributing to neuroinflammatory processes, while Ang II receptor type 1 has been found to be highly expressed in brain areas involved in the HPA axis, as well as the sympathoadrenal system (Saavedra and Benicky, 2007). Therefore, both the vascular and cerebral effects of ET-1 and Ang II could contribute to CAD and depression/anxiety comorbidities. Indeed, Zhou et al. analyzed 3154 stable CAD patients for 24 months; they were divided into event and non-event groups, depending on whether adverse cardiovascular events occurred. The event group, compared to non-event, had higher circulating “big ET-1”, the precursor of mature ET-1, which was positively associated with adverse cardiovascular outcomes (Zhou et al., 2017). In line with these findings, Fan et al. found that patients with poor coronary collateral circulation had significantly higher plasma ET-1 than those with good coronary collateral circulation (Fan et al., 2016). Comparable results were observed for Ang II by Li et al. (Li et al., 2016) in Watanabe heritable hyper-lipidemic rabbits. There, rabbits infused with high Ang II levels (200 ng/min/kg), compared to those infused with low Ang II (100 ng/min/kg) or saline vehicle, had the highest mortality rates, at 92 %, compared to 50 % in low Ang II and 0 % in vehicle. This is likely due to the elevated Ang II levels destabilizing the coronary plaques, subsequently triggering thrombosis (Li et al., 2016). Therefore, endothelial injury is an important factor behind atherosclerosis and CHD pathogenesis.

Endothelial dysfunction markers have also been found to be associated with anxiety and depression (Tonhajzerova et al., 2020), in which depressed patients had significantly increased levels for endothelial damage-related markers (Geraets et al., 2020; Lesperance et al., 2004). In fact, Burg et al. found that depressive symptom severity in CHD patients is related to ET-1 levels (Burg et al., 2011). Additionally, vascular endothelial growth factor (VEGF), an important bioactive substance secreted by ECs, has been associated with the development of depressive-like behaviors under stress, likely via increasing blood-brain barrier permeability (Matsuno et al., 2022). However, the putative association between VEGF levels and behavioral outcomes, whether it is correlation or causation, should be taken with caution.

The endothelial injury process also involves exosomes, which could induce endothelial injury and barrier dysfunction, leading to coronary disease progression. For instance, Wang et al. found that human smokers had greater exosome counts, along with higher miR-155 levels, compared to non-smokers; these smokers also had carotid plaque formation under ultrasound imaging. Furthermore, injecting nicotine-induced mouse exosomes, secreted from monocytes, into Apoe^−/−^ mice exacerbated atherosclerosis progression, which may be owed to increased exosome miR-155 activity in monocytes. Indeed, mouse exosomes carrying miR-155 could elicit EC dysfunction by activating NF-κB signaling, while this effect is attenuated by inhibiting miR-155 with GW4869 (Wang et al., 2023). Along similar lines, Li et al. also observed that circulating exosomes, isolated from patients with traumatic brain injury, could trigger endothelial dysfunction and result in secondary brain injury (Li et al., 2023). Therefore, anxiety/depression could aggravate endothelial injury, possibly by stimulating the release of some mediators in exosomes, as well as increasing endothelial damage proteins, such as ET-1, to exacerbate vasoconstriction, thrombosis, and coronary disease progression. Thus, exosomes play an essential role in cardiac dysfunction via endothelial injury and impairment.

## 3 The application of exosomes for treatment

Owing to the roles that exosomes play in physiological and pathophysiological processes, including CHD with depression/anxiety, the manipulation of these exosomes for therapeutic applications has become a topic of great interest. Indeed, multiple studies have found exosomes to be a versatile tool for translational medicine, such as for disease diagnosis, treatment, and prevention (He et al., 2018). This is owed to them being rich in diagnostic/prognostic biomarkers, along with them being easily obtainable, stable in body fluids, and possessing disease-specific cargos (Wang et al., 2021). For instance, Son et al. have found that patients with chronic total occlusion (CTO), which has better short-term, but worse long-term outcomes compared to those with acute MI (AMI), could be distinguished from AMI via differences in exosome miRNA contents and expression levels. In particular, miR-9-5p and miR-127–3p were significantly lower in CTO versus AMI patient exosomes, demonstrating that exosome miRNAs could serve as biomarkers to differentiate between the 2 diseases (Son et al., 2024). Likewise, pro-inflammatory miRNA levels in the exosomes of peripheral artery disease patients were found to correlate with atherosclerosis severity (Sorrentino et al., 2020). These findings thus illustrate the significant prognostic/diagnostic biomarker potential for exosomes and their miRNA contents. However, their widespread clinical applicability is still limited by the lack of validation in large cohort studies and the International Organization for Standardization (Ayers et al., 2019).

Additionally, a large number of studies have been conducted on applying exosomes for treating CHD, as evidence suggests that they are able to improve endothelial damage, promote vascular regeneration, along with serving as drug, therapeutic protein, or miRNA carriers (Xuan et al., 2022). Moreover, in recent years, a variety of cardio-protection-related exosome miRNAs, such as miR-218, miR-128–3p, and miR-143, have received widespread attention (Gao et al., 2020; Farina et al., 2020; Wang et al., 2020). Specifically, miR-218 is a crucial regulator, whose overexpression promotes angiogenesis ability, induces apoptosis, and alleviates inflammatory injuries (Gao et al., 2020), while miR-128–3p is a critical modulator of vascular smooth muscle cells (Farina et al., 2020); by contrast, it inhibits cardiomyocyte proliferation and function. As for miR-143, it is similar to miR-128–3p, in that it serves as a key regulator of cardiac development, along with being involved in vascular smooth muscle cell function. Therefore, exosomes could potentially be administered to improve cardiovascular function in CHD patients, via the delivery of various cardio-protective components, such as miRNA.

Exosomes have also been a source of great interest for treating anxiety and depression, as they have been documented to exert neuroprotective effects. For instance, intestinal microbiota-secreted exosomes lowered ethanol-affected exosome-induced anxiety-like behavior and neuro-inflammation in a probiotic gavage mouse model (Pei et al., 2024). Neuroprotective effects were also observed for astrocyte exosomes (Jin et al., 2024), likely via their modulation of inflammation and glial cell activities to enhance damaged neural tissue function (Jin et al., 2021). Such modulation involved regulating microglia activation (Ding et al., 2018), inhibiting the NLRP3 inflammasome, via targeting programmed cell death 4 with exosome miR-183–5p (Ding et al., 2021), down-regulating cyclin-dependent protein kinase 5-related nigrostriatal autophagy (Li et al., 2021), as well as inhibiting pro-inflammatory mediator upregulation (Kaniowska et al., 2022), thereby reducing neuro-inflammation. With respect to depression, natural killer cell exosomes with miR-207 could alleviate depressive symptoms in mice by targeting TNF-related apoptosis-inducing ligand to inhibit astrocytic NF-κB signaling (Li et al., 2020), while exosomes derived from bone marrow MSCs could improve hippocampal neuron damage in depressive rats by upregulating miR-26a (Guo et al., 2020). Overall, exosomes could be used to alleviate anxiety and depression, owing to their contents being able to exert neuroprotective effects. Their ease in crossing blood vessel walls and biological barriers bestow them with excellent biocompatibility and bio-distributive capabilities (Zhang et al., 2020), which has led to them gaining significant research interest (He et al., 2018) with respect to CHD and depression/anxiety treatment (Table 2). However, clinical applications of exosomes remain challenging due to inadequate targeting specificity and lack of standardized, large-scale production (Rezaie et al., 2022). Nevertheless, exosomes are still a promising therapeutic approach for CHD with depression/anxiety.

**Table 2:** Studies regarding the application of exosomes for treating diseases.

Author	Study Model	Therapeutic Contents	Research subject
Wang et al. (2021)	Animal experiment	miR-1246	Rats
Xuan et al. (2022)	Animal experiment	Bone morphogenic protein 2	Rats
Ding et al. (2021)	Animal experiment	miR-183–5p	Rats
Kaniowska et al. (2022)	Animal experiment	miR-188–3p	Mice
Li et al. (2020)	Animal experiment	miR-207	Mice
Guo et al. (2020)	Animal experiment	miR-26a	Rats

## 4 Conclusion and perspectives

Anxiety and depression are important independent CHD risk factors, and the associations between psychological factors and heart disease has become a topic of great interest. In this review, we outline the current research on the pathogenesis of CHD with anxiety/disorder, as well as potential treatment strategies, with focus on exosomes. The main mechanisms that are likely behind the comorbidity of CHD with anxiety/depression may include inflammatory responses, neuroendocrine signaling abnormalities, altered SNS regulation, platelet activation, and endothelial injury. In all of these processes, exosomes play key roles in CHD, anxiety, and depression pathogenesis. Consequently, examining the role of exosomes in CHD with concurrent anxiety/depression may be greatly beneficial for formulating effective treatment strategies for those patients. However, there is still a lack of basic or large-scale clinical studies on patients with CHD and anxiety/depression; more studies are thus required to fully elucidate the link between these diseases.

Exosomes have been investigated for numerous clinical applications, such as biomarkers for disease prognostication and diagnoses, as well as drug delivery carriers and therapeutic targets. However, their utilization for these applications have significant challenges and limitations, necessitating further investigations. One significant challenge is the methods used for exosome isolation, which could critically impact the accuracy and clinical utility for subsequent analyses. As a result, standardizing isolation and analysis techniques could contribute to advances in exosome clinical applications. Another significant challenge behind widespread exosome application is that additional translational studies, among different patient demographics, are required. Nevertheless, the clinical potential of exosomes remains untapped. Future efforts could thus involve the development of exosome biomarkers, therapeutic strategies targeting exosome-mediated pathways, as well as standardizing protocols for isolation and analyses, all of which could provide insights to the clinical value of exosomes, and in turn, provide better treatment options for patients.

## CRediT authorship contribution statement

**Jiecheng Huang:** Writing – review & editing, Writing – original draft, Conceptualization. **Ying Piao:** Writing – review & editing, Writing – original draft, Conceptualization. **Jingjin Liu:** Writing – review & editing, Writing – original draft, Conceptualization.

## Ethical statement

An ethical statement is not applicable for this article.

## Funding

This work was supported by the National Natural Science Foundation (Project # 82200315), Sanming Project of Medicine in Shenzhen (No. SZSM201412012), and Major Scientific Research Project of Shenzhen People's Hospital (SYWGSJCYJ202301).

## Declaration of competing interest

The authors declare no conflicts of interest.

## Data Availability

No data was used for the research described in the article.
